# Malignant Melanoma in the Groin Masquerading as a Vascular Tumour: A Rare Cytodiagnosis

**DOI:** 10.7759/cureus.69991

**Published:** 2024-09-23

**Authors:** Shreya Giri Goswami, Arvind Bhake, Poornima Pandey

**Affiliations:** 1 Pathology, Jawaharlal Nehru Medical College, Datta Meghe Institute of Higher Education and Research, Wardha, IND

**Keywords:** fnac, inguinal mass, metastasis, metastatic malignant melanoma, metastatic melanoma, vascular tumor

## Abstract

Malignant melanoma (MM) is an aggressive cutaneous neoplasm tending to metastasise anywhere within the body. It frequently metastasises to the draining lymph nodes. A distinct presentation of MM occurs when metastatic lesions appear in the regional lymph nodes, while the primary tumour remains clinically undetected. Fine needle aspiration cytology (FNAC) is highly sensitive, rapid, and cost-effective. The utility of the FNAC procedure is well established in the patient's diagnostic workup. However, the Indian data on the cytodiagnosis and cytomorphology of metastatic MM are very scarce. We submit a clinicopathological profile of a rare case in a 68-year-old male, where FNAC from an inguinal mass led to the diagnosis of metastatic MM, which mimicked clinically as a vascular tumour. The present case highlights MM's unusual clinical presentation and cytomorphological details through FNAC.

## Introduction

Among all malignant neoplasms, melanoma is rare, accounting for 1-3% of all malignancies worldwide [1-4]. It is a highly aggressive neoplasm caused by excessive proliferation of abnormal melanocytes and has an increasing incidence worldwide [5]. It tends to metastasise through lymphatic and hematogenous routes early in the disease process. It mostly involves the head, neck, and lower extremities [3]. It frequently metastasises to the regional lymph nodes. Patients with metastatic melanoma have poorer survival rates [2]. The overall five-year survival rate (OS) is 23% for advanced melanoma and 98% for early melanoma, respectively [5]. In some cases, metastatic lymphadenopathy of malignant melanoma (MM) is a primary presenting lesion, even in the absence of any other lesion. The metastatic lesions can be misdiagnosed as primary locoregional tumours on clinical-radiological assessment [1,3,4].

The utility of fine needle aspiration cytology (FNAC) diagnosis for the initial evaluation of metastatic lesions is well established [6]. In such cases, FNAC is a useful procedure for yielding neoplastic cells for the diagnostic assessment of the lesion. Attempts have been made to describe the cytomorphological features of metastatic melanoma and to help assert the preoperative diagnosis, but the Indian literature on this topic is scarce. This report is submitted to highlight the unique clinical presentation and cytomorphological features through FNAC. The case was diagnosed as a tumour of vascular origin on clinical assessment, but further evaluation through FNAC provided more detailed insights into its nature.

## Case presentation

**Figure 1:**
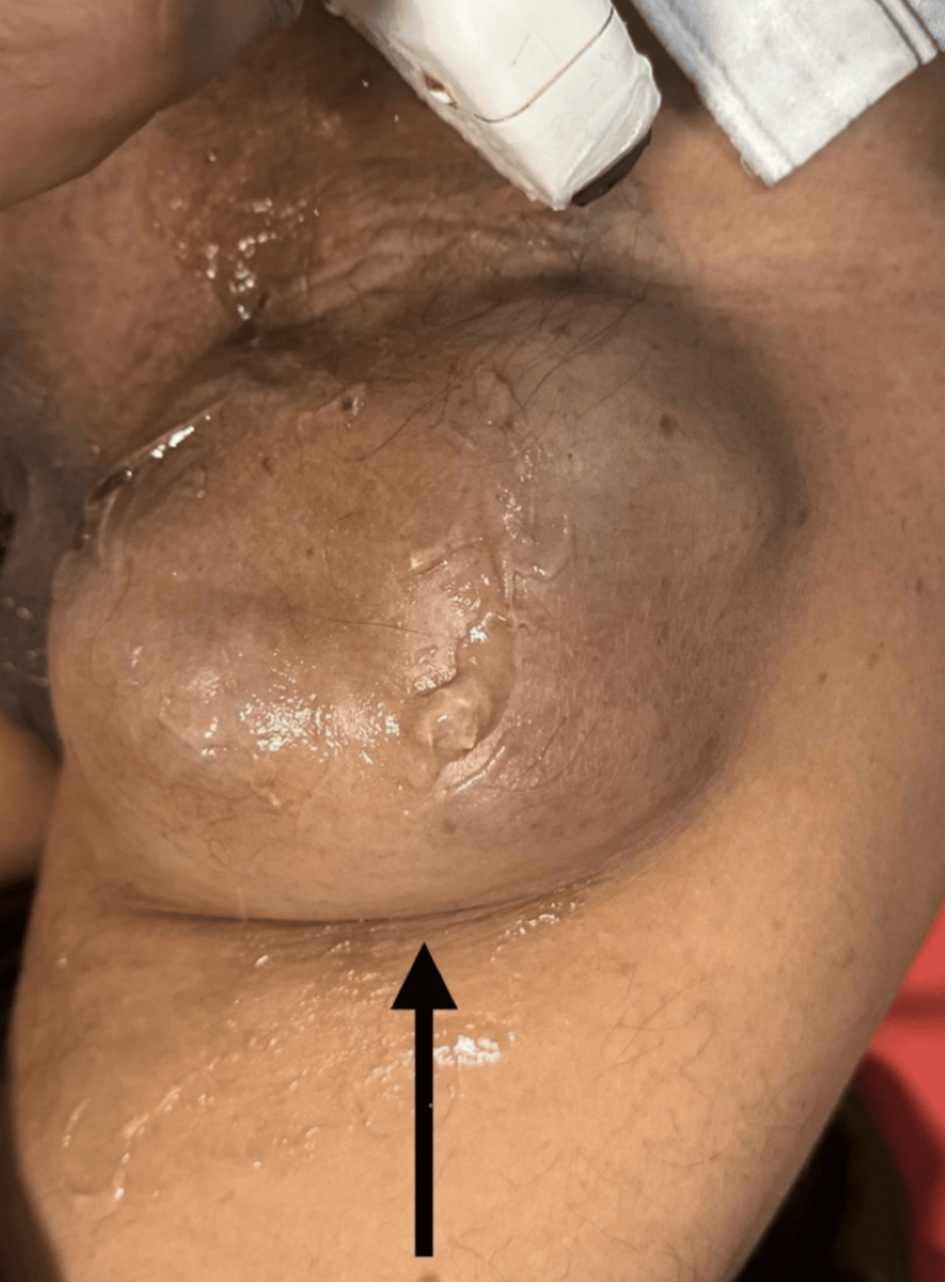
Clinical photograph of nodular swelling (black arrow) in the left inguinal region.

A 68-year-old male patient presented in the Outpatient Department of Surgery at the tertiary care hospital with complaints of swelling and pain over the left inguinal region for seven months. The swelling was gradually progressive and was associated with pain. The nature of the pain was dull and aching (Figure 1).

**Figure 2:**
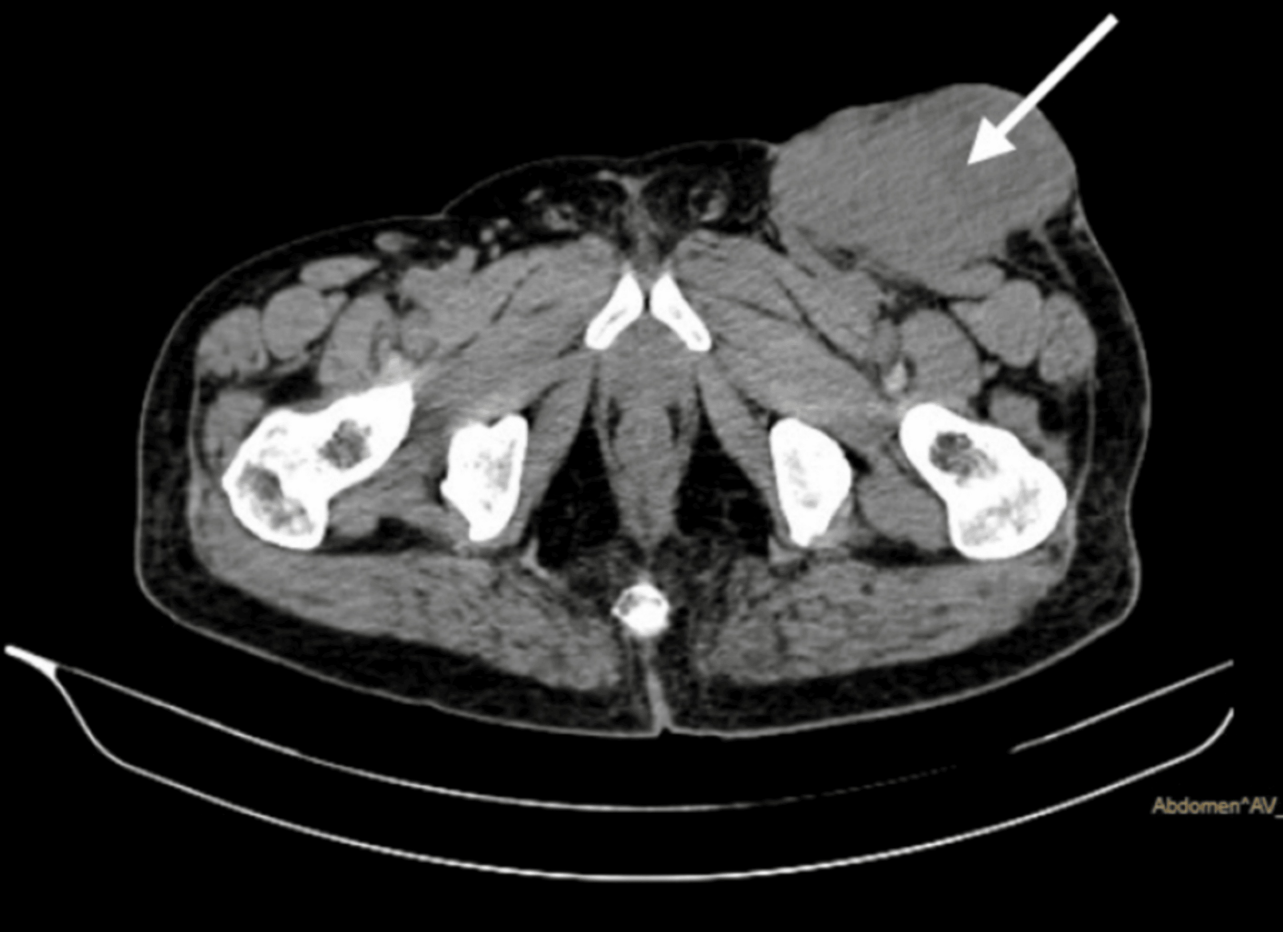
Contrast-enhanced computed tomography - pelvis shows a well-defined hyperdense soft tissue lesion (white arrow) in the left inguinal region.

The patient did not provide any history of locoregional trauma. He had provided a history of left foot amputation around one year ago. The histological diagnosis of the amputated foot was unavailable. A preliminary clinical diagnosis identified the tumour as likely being of vascular origin. The rest of the general and systemic examination was normal. The patient's blood profile and coagulation studies revealed no abnormalities. The ultrasonography (USG) of the locoregional site revealed a multilobulated, heterogeneously enhancing hyperechoic mass lesion with rich vascularity in the subcutaneous plane of the left inguinal region. The contrast-enhanced computed tomography (CECT) of the thorax and pelvis revealed a well-defined, hyperdense soft tissue density lesion in the left inguinal region at the subcutaneous plane (Figure 2).

**Figure 3:**
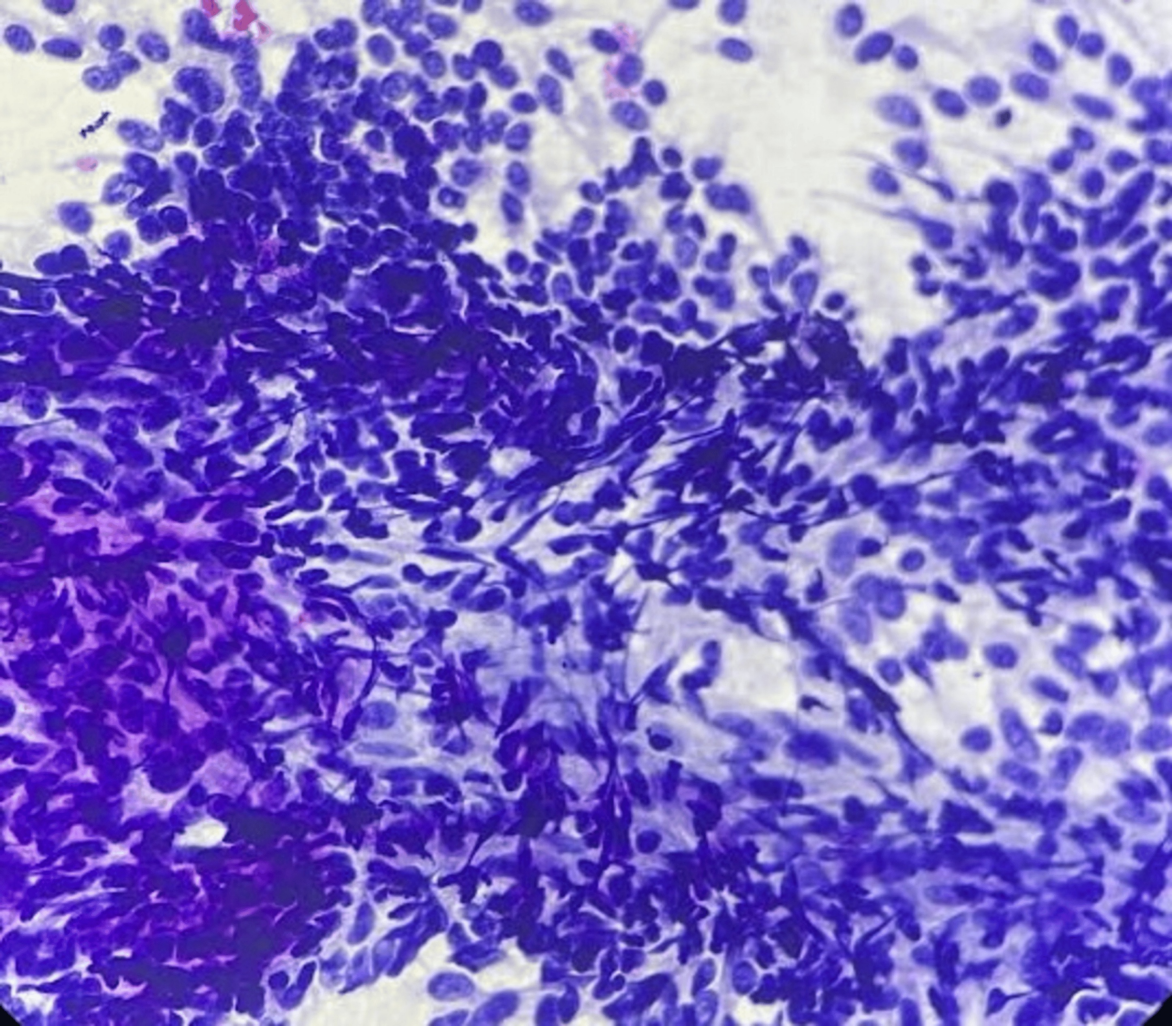
Photomicrograph of fine needle aspiration smears from inguinal mass shows sheets of small polygonal and spindle like epithelial cells (Papanicolaou stain, 40x).

The radiodiagnosis of a primary neoplastic lymph nodal mass was offered, and FNA correlation was advised. The patient underwent FNA under USG guidance from the swelling in the left inguinal region. On assessment, the swelling in the left inguinal region was large and nodular, measuring 11.5 x 10 cm. It was tender, firm in consistency, and fixed, with feeble pulsations. The overlying skin was tense, showing prominent small veins, without a local temperature rise. The material from the FNA yielded dark brown blood-mixed material. This material was smeared on glass slides and stained with May-Grünwald Giemsa and Papanicolaou stains. The smears revealed a few polygonal intermediate-sized cell sheets and the presence of sheets of spindle-like epithelial cells with poor cellular cohesion (Figure 3).

**Figure 4:**
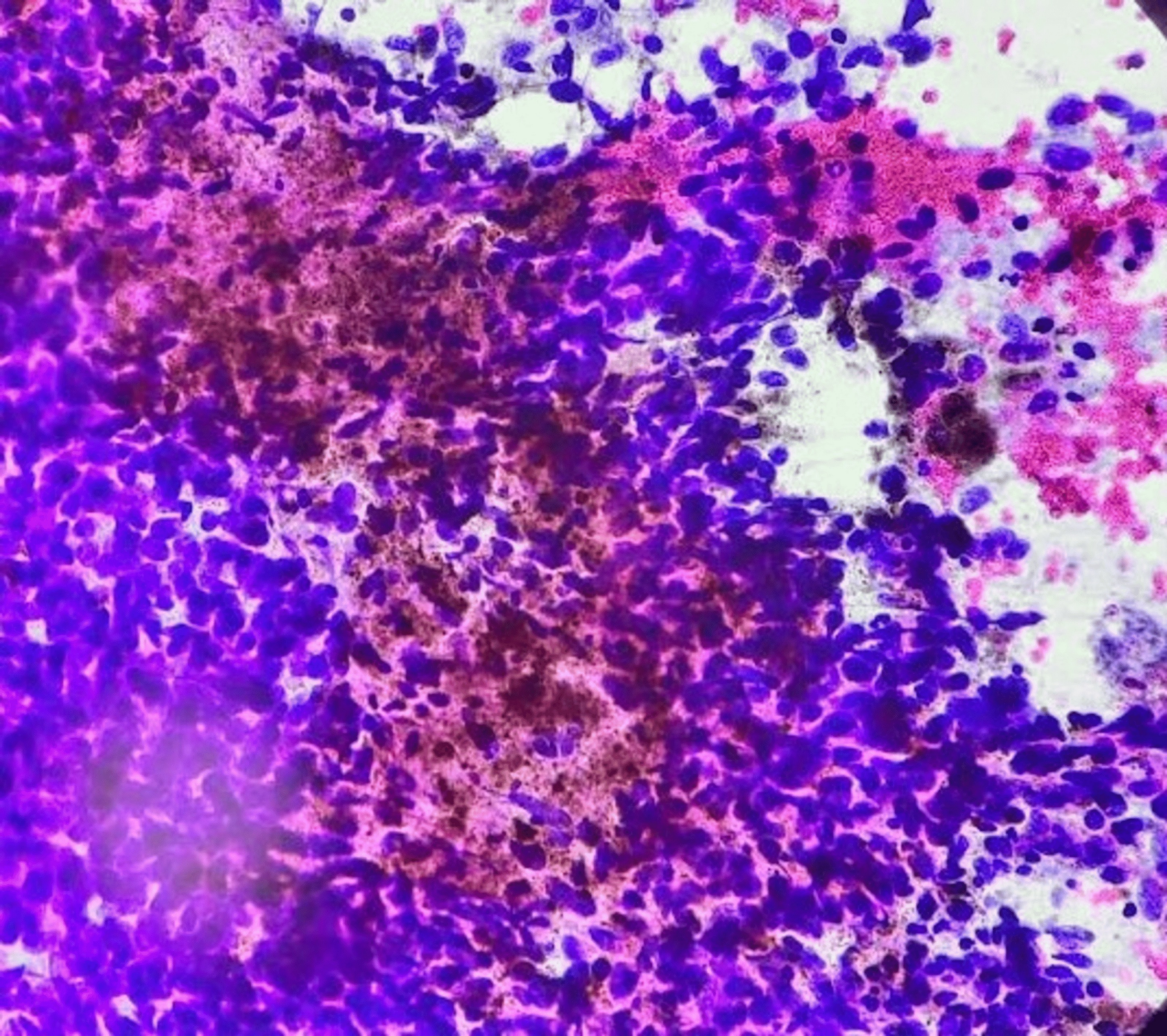
Photomicrograph of fine needle aspiration smears from inguinal mass: cytoplasm of the few cells shows brown granular melanin pigment (Papanicolaou stain, 40x).

These cells carried hyperchromatic, centrally placed, enlarged, pleomorphic nuclei with nucleoli. The cytoplasm of a few cells showed brown granular pigment. In the background of the smear were melanin-laden macrophages in isolated places (Figure 4).

**Figure 5:**
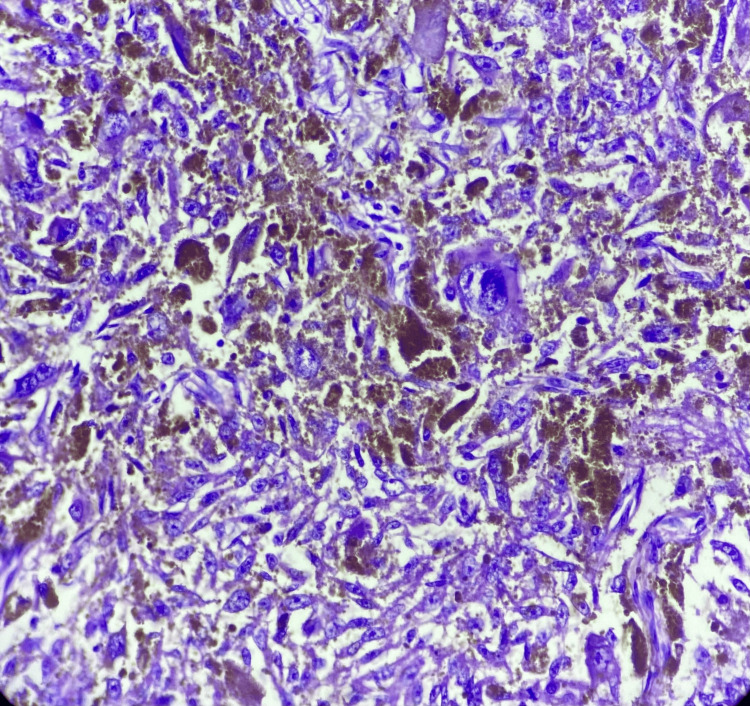
Photomicrograph of metastatic melanoma of inguinal mass, section shows spindle-shaped cells with severe nuclear atypia. Brown pigments are also seen (hematoxylin and eosin stain, 40x).

The cytodiagnosis of deposits of the spindle cell variant of MM was offered. Following the cytodiagnosis, the tumour board of the institute advised the patient to undergo wide local excision of the mass. The patient underwent wide local excision of the left inguinal mass, and the specimen was sent for histopathological examination. The histomorphology of the resected specimen revealed a scattered population of spindle-shaped, pleomorphic tumour cells. These cells contain scant basophilic cytoplasm with hyperchromatic nuclei containing prominent nucleoli and intracytoplasmic brown granular melanin pigment. Histological features were suggestive of metastasis of MM (Figure 5).

**Figure 6:**
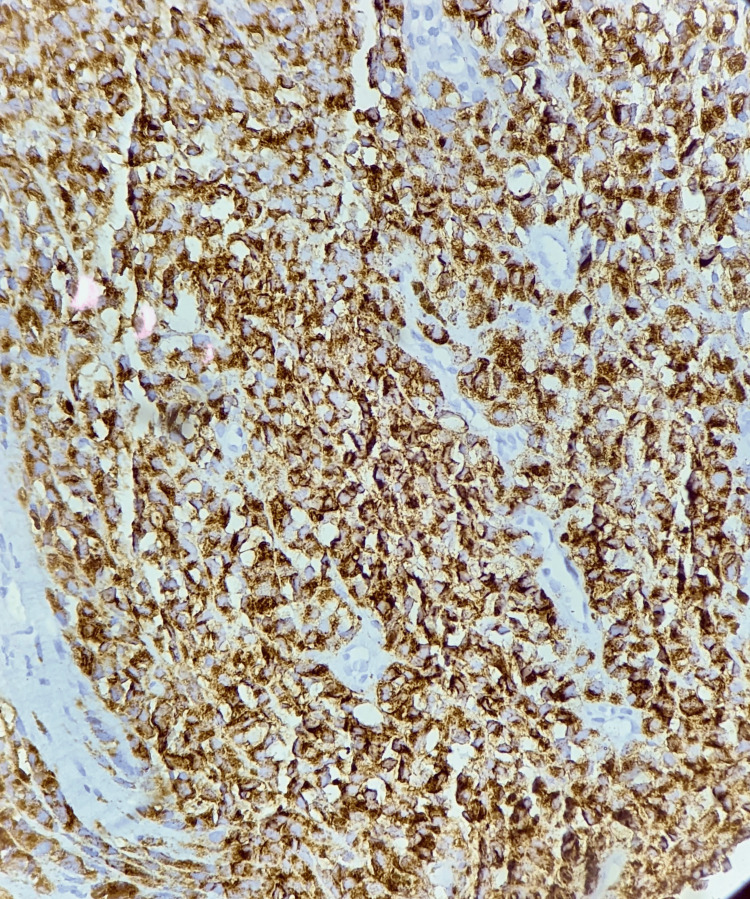
Immunohistochemistry - human melanoma black 45 (HMB-45), malignant melanoma: photomicrograph shows cytoplasmic immunoreactivity (IHC: HMB-45, 40x).

Immunohistochemistry for human melanoma black-45 (HMB-45) (Figure 6) and S-100 was applied, and these were confirmatory for MM. No other lymph nodes were involved, nor was any distant metastasis present. The patient continued to receive follow-up care under the tumour board of the institute.

## Discussion

MM is a highly aggressive tumour known for its widespread metastasis, most frequently to lymph nodes, subcutaneous tissue, or viscera [1,7]. Metastatic melanoma with unknown primary presents in 2.3-16.6% of patients [7]. Up to 90% of melanomas exhibit abnormal activation of the mitogen-activated protein kinase (MAPK) pathway, leading to cell cycle disruption and inhibition of apoptosis. The most common genetic alteration in melanomas is a mutation in the B-raf proto-oncogene (BRAF) gene [3]. The most commonly involved nodal groups are axillary, cervical, and inguinal nodes [6,7]. A study conducted by Radhika et al. [2] on 30 patients diagnosed with melanoma on FNAC revealed the inguinal lymph node as the most common site of secondary involvement, followed by submandibular and axillary lymph nodes. Other sites of metastasis included the liver, lung, and abdomen [2]. However, a study by Ghartimagar et al. [6] revealed the cervical triangle as the most common site of metastatic involvement. As per the literature, the age group affected ranges from 24 to 86 years, with a mean age of presentation at 57.5 years [5,6,8]. Metastatic MM most commonly affects women over men, with a 2:1 ratio [5-8]. However, the presented case of inguinal nodal metastasis of melanoma was of a 68-year-old male, similar to the studies of Prakash et al. [3], Chakrabarti et al. [1], Sahu et al. [4], and Mehrotra et al. [9]. A single-centre study conducted by Radhika et al. [2] included a total of 30 patients diagnosed with melanoma on FNAC, consisting of 19 males and 11 females, with an M:F ratio of 1.7:1. Ronchi et al. [10] stated that younger patients aged 20-24 years tend to have a higher prevalence among females, while males are more commonly affected in those over 55 years of age.

The diagnosis of metastatic melanoma was not straightforward in this case, as the primary tumour remained elusive. The probable reason for the amputation of the foot in this case may be the presence of plantar MM. Upon clinical evaluation, Sahu et al. [4] noticed the inguinal swelling as nodular, smooth, and glossy. It described the swelling as multiple, smooth, shiny, pigmented nodular lesions with verrucosity and ulceration on the surface. A differential diagnosis of Kaposi sarcoma and angiosarcoma was considered by Sahu et al. [4]. The present case observed a similar differential diagnosis of a tumour of vascular origin. The FNAC material yielded from metastatic lymph nodes was dark brown pigmented material. The characteristic appearance of FNAC material in the case of MM has also been described by Radhika et al. [2] and Prakash et al. [3]. The cytomorphological characteristics of metastatic melanoma are distinctive and are less frequently documented in Indian literature. Radhika et al. [2] studied the cytomorphology of metastatic melanoma in 30 patients and described the common features as (i) clusters of epithelioid and spindle cell patterns of tumour cells, (ii) these cells contain granular cytoplasm, centrally placed nuclei with condensed chromatin and prominent nucleoli, (iii) pigmented melanophages were seen, (iv) few cells contain melanin pigment, and pseudo-inclusions were seen in a few, (v) binucleated cells, along with mitotic figures, were also seen.

Chakrabarti et al. [1], Prakash et al. [3], and Chaudhary and Patni [11] observed similar morphological features in the aspirates of metastatic MM. The features were elaborated as follows: (i) groups and sheets of pleomorphic round tumour cells; (ii) these cells have abundant basophilic cytoplasm with pleomorphic and hyperchromatic nuclei containing prominent nucleoli; (iii) cells contain black granular pigment in the cytoplasm. The cytomorphological features observed in the present case were similar to the findings of the aforementioned studies. The histomorphology of the given specimen was consistent with the cytodiagnosis of metastatic MM.

## Conclusions

The literature on the cytological diagnosis of metastatic MM in the Indian population is very scarce. Therefore, the case report was submitted for its unique and rare clinical presentation and its distinct cytomorphological features, which clinically mimicked a vascular tumour upon evaluation. The case is unique, as FNAC has helped in the diagnosis of the metastatic spindle cell variant of MM in a patient with a surgical amputation of the foot.
